# The microbiome of modern microbialites in Bacalar Lagoon, Mexico

**DOI:** 10.1371/journal.pone.0230071

**Published:** 2020-03-25

**Authors:** Alfredo Yanez-Montalvo, Selene Gómez-Acata, Bernardo Águila, Héctor Hernández-Arana, Luisa I. Falcón

**Affiliations:** 1 UNAM, Instituto de Ecología, Parque Científico y Tecnológico de Yucatán, Sierra Papacal, Yucatán, México; 2 El Colegio de la Frontera Sur Unidad Chetumal, Chetumal, Quintana Roo, Mexico; The University of Akron, UNITED STATES

## Abstract

Microbialites are highly diverse microbial communities that represent modern examples of the oldest life forms, stromatolites (dated >3.7 Ga). Bacalar Lagoon, in Mexico, harbors the largest freshwater microbialite occurrences of the world; yet diverse anthropogenic activities are changing the oligotrophic conditions of the lagoon. The objective of this work was to perform a spatial exploration of the microbialites of Bacalar Lagoon, analyze their prokaryote diversity, following a high throughput sequencing approach of the V4 region of the 16S rDNA, and correlate to the environmental parameters that influence the structure of these communities. The results indicate the presence of microbialites throughout the periphery of the lagoon. The microbiome of the microbialites is composed primarily of Proteobacteria (40–80%), Cyanobacteria (1–11%), Bacteroidetes (7–8%), Chloroflexi (8–14%), Firmicutes (1–23%), Planctomycetes (1–8%), and Verrucomicrobia (1–4%). Phylogenetic distance analyses suggests two distinct groups of microbialites associated with regions in the lagoon that have differences in their environmental parameters, including soluble reactive silicate (in the north), bicarbonates and available forms of nitrogen (ammonium, nitrates and nitrites) (in the south). These microbialite groups had differences in their microbiome composition associated to strong anthropogenic pressure on water quality (agriculture, landfill leachate, lack of water treatment infrastructure and intensive tourism), which were related to a loss of microbial diversity.

## Introduction

Bacteria and Archaea (prokaryotes) represent the most diverse and abundant organisms on the planet **[1].** They are involved in maintaining and controlling biogeochemical cycling of the fundamental elements of life (H, C, N, O, S and P) **[2].** Understanding the multiple ecological and evolutionary processes that are related to the distribution and structure of prokaryote diversity at the local and global scales is a main interest of microbial ecology **[3–4]**. The formation of biogeographic distribution patterns in prokaryotes is determined by environmental heterogeneity (ecological factor) and dispersion (historical factor) **[5].** At the local scale, factors that include pH, habitat heterogeneity, system productivity, and more recently, human alteration of the habitat, are contributing to shape prokaryote diversity and structure **[6–8].** Industrial activities which modify land use including agriculture, mining and wastewater discharges cause direct changes in the structure of microbial communities **[9–10]**. Studies based on environmental DNA sequencing suggest that prokaryotes are biological monitors of anthropogenic environmental change **[11–12]**.

Knowledge of the factors that define communities, including the interactions that shape community structure and dynamics, within a certain environmental matrix, are fundamental to understand shifts related to habitat transformation **[13].** Ecological analysis based on the spatial distribution of diversity (α, β, γ) **[14]** is the basis to defining the emergent properties of communities **[15]**, and a relevant tool to monitor ecosystem function **[16–18]**.

Microbialites are diverse microbial communities that precipitate carbonates, silicates and sulfate minerals, through the interaction of their metabolisms with the environment **[19–23]**. Fossil microbialites (stromatolites) have been dated in ~3.5–3.7 Ga years **[24–26]** and represent the oldest evidence of life on Earth. Microbialites are present in modern aquatic environments, both freshwater and marine. Microbialites can be found in saline marine environments such as the Hamelin Pool of Shark Bay (Western Australia), Cayo Cocos (Cuba) and in Highborne Cay (Bahamas); in lacustrine environments including Pavilion Lake and Clinton Creek (Canada), Lake Tanganyika (Africa), Lake Salda Golu (Turkey), Cuatro Cienegas and Lake Alchichica (Mexico), Ruidera Pools (Spain) and Great Salt Lake (GSL) (United States) **[13, 22–23, 27, 83],** among others.

The genetic composition of microbialites has been studied with different approaches, and Proteobacteria, Cyanobacteria, Actinobacteria, Bacteroidetes and Chloroflexi are their main constituents **[22, 27]**. Moreover microbialites from Pavilion lake have a high abundance of Proteobacteria (Alphaproteobacteria and Deltaproteobacteria) and Acidobacteria, principally photoheterotrophic *Rhodobacter*, *Rhodomicorbium*, *Phodopseudomonas* and *Rhodospirrillum*, heterotrophic *Sphingomonas*, nitrogen-fixing *Bradyrhizobium* and *Rhizobium*, dissimilatory sulfate reducing *Desulfobacterium* and *Desulfovibrio*, heterotrophic *Myxococcus*, Cyanobacteria such as *Anabaena*, *Lyngbya*, *Nostoc* and *Oscillatoria* **[22]**. While microbialites from hypersaline Storr’s lake (Bahamas) have high abundance of Chloroflexi, Deltaproteobacteria and Spirochaetes **[28].** Microbialites from Great Salt Lake are dominated by Alteromonadales, Oceanospirillales, Flavobacterales, Cytophagales, Chlorococcales and Chromatiales, with archaeal represented by *Halorubrum* sp., Halobacterales and Haloferacales **[13].** In Mexico, there are several environments that harbor microbialites which share similar genetic composition at the phylum level, although each microbialite is different at the species level. We now know that microbialites in Mexico show differences in their genetic composition related to geographic region and that conductivity, concentration of nitrate and temperature are among the variables that structure their composition [8].

Microbialites constitute complex communities in which all pathways needed for biomass formation and recycling are present. Nitrogen fixation associated to heterocystous cyanobacteria, which can couple this pathway with oxygenic photosynthesis, is a fundamental metabolism in microbialites **[29–30]**. Cyanobacteria are fundamental microbialite builders, through the coupling of photosynthesis, nitrogen fixation and Extracellular Polymeric Substance (EPS) matrix synthesis **[31–32]**. Aerobic and anaerobic heterotrophic bacteria are associated with the cyanobacterial biofilm and contribute to biomass cycling **[22, 33]**; further, the role of sulfur-bacteria has been related to mineral precipitation in microbialites **[34–36]**.

Bacalar Lagoon has been documented as the largest freshwater microbialite ecosystem in the world **[37–38].** Several authors have studied Bacalar microbialites [23, 30, 37–39], but have focused on specific areas of the lagoon. Bacalar microbialites have been described as actively fixing N_2_ during the daytime [30], and harbor a vast diversity of cyanobacteria and sulfur bacteria [38–39]. In this study we wanted to answer if habitat transformation of Bacalar Lagoon influences microbialite community structure and composition. We characterized the microbiome of Bacalar microbialites throughout the lagoon and analyzed if there are structuring effects on their prokaryote composition related to environmental variables, following a next-gen sequencing approach of the V4 hypervariable region of the 16S rDNA gene.

## Materials and methods

### Study site

**Fig 1:**
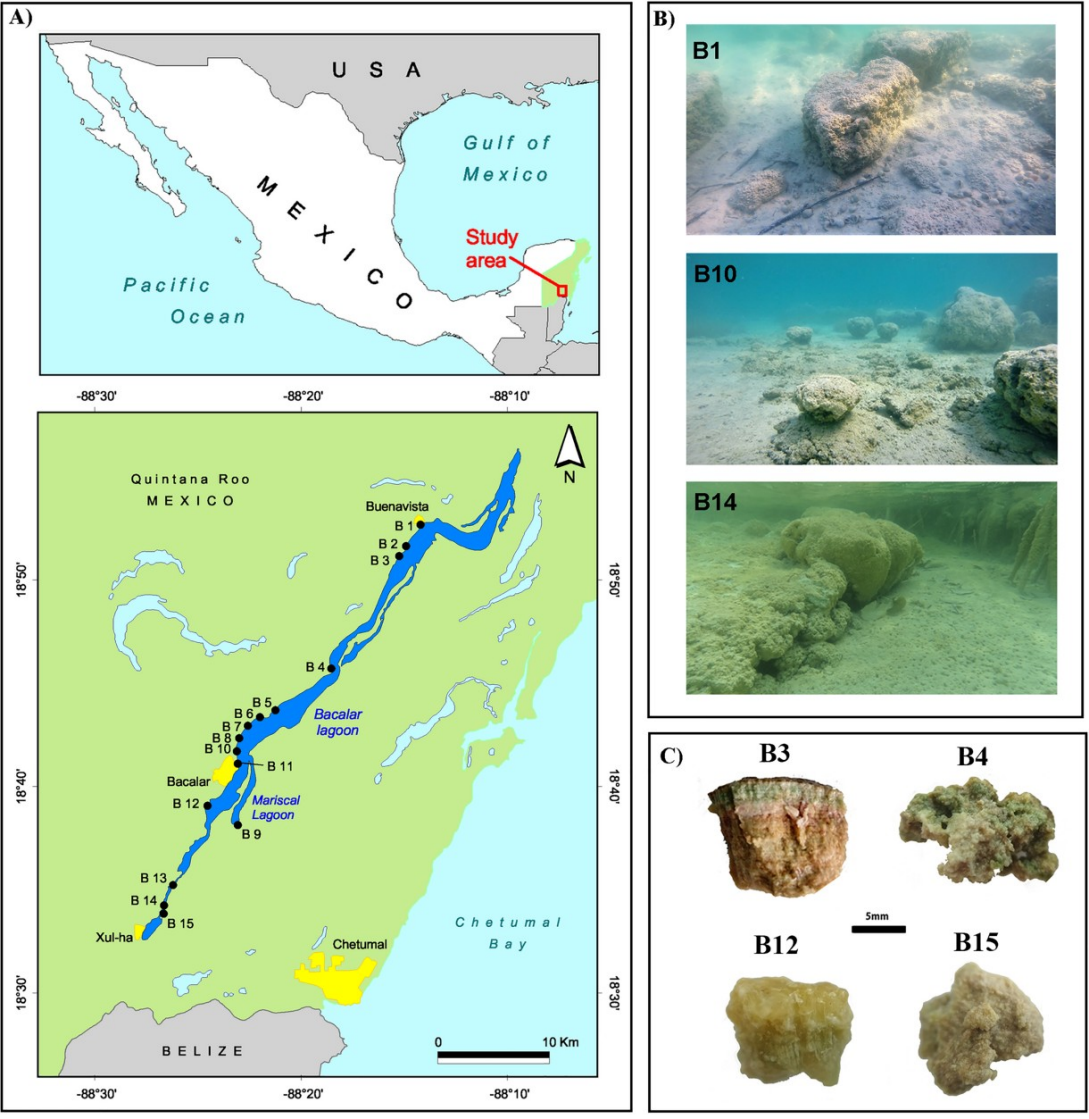
a) Location of Bacalar Lagoon, Mexico and microbialite sampling sites for this study; b) Example of morphology of microbialites from Bacalar Lagoon; c) cross-section of microbialites from Bacalar Lagoon, indicating sampling sites.

Bacalar is a karstic and freshwater lagoon located in the southeast of Quintana Roo, Mexico in the Yucatan peninsula (Fig 1, S1 Table). The lagoon is a geological fault due to its orientation and shape. Bacalar Lagoon, has been considered an oligotrophic system due to the low concentration of nitrogen (N) and phosphorus (P), and is part of the Transverse Coastal Corridor, a complex water system, where a series of karst freshwater lakes, lagoons and estuaries are connected through underground water flows **[40]**. Temperature and pH range between 28–31°C and 7.7–8.2, respectively **[27, 39, 41]**. Hydrogeochemistry is characterized by higher concentration of calcium (Ca^2+^) **[37]** and sulfate (SO_4_^2-^), compared to other karstic lagoons in the south of the Yucatan peninsula **[39]**. Bicarbonate concentration (HCO_3_^-^) in southern Bacalar Lagoon, is higher than marine levels, due to the presence of five sinkholes (locally known as “cenotes”) that are sites of groundwater intrusion to the lagoon **[37–38, 41].** Bacalar Lagoon has a north-south and south-north water circulation pattern, that converges towards the middle of the lagoon, and flows towards the Bay of Chetumal to the East **[38]**.

### Sample collection

Microbialites were collected in 15 sites along the western shore of Bacalar Lagoon along a north-south gradient. Cores of approximately 2.5 cm in diameter were sampled in duplicates from individual microbialite heads, and three to five individuals per site were sampled (Fig 1, S1 Table). Samples were taken with gloves and sterile material to avoid cross-site contamination. Collection was carried out during the spring of 2018. Samples were stored at 4°C during transport to the laboratory where they remained frozen at -70°C until processed. All microbialite samples were carried under collector permit PPF/DGOPA-113/14 awarded by SEMARNAT, Mexico. Field studies did not involve endangered or protected species.

Three water samples (500 mL) were taken at each sampling point using Nalgene bottles, previously washed with 15% HCl, and were filtered (0.22 μm Millipore membrane) *in situ* and stored at 4°C for dissolved nutrients analysis. *In situ* conductivity, pH and temperature were measured using a YSI Professional handheld (YSI model Pro 30) and pH-meter (Hanna HI 9146).

The degree of tourist visitation per site was assessed during the sampling with interviews to locals. A high level represents sites that have tourism throughout the year; medium represents sites that only have tourists during holiday seasons; low represent sites that are seldom visited by tourists.

### Nutrient analysis and statistical analysis of environmental variables

Nutrient measurements were done with colorimetric methods using a UV-visible spectrophotometer (SHIMADZU, Model UV-1700). Ammonium, NOx (nitrites and nitrates), soluble reactive silicate (SRSi) and soluble reactive phosphorus (SRP), were analyzed **[42–43].** All analyses were performed in triplicate in the Chemistry Laboratory at ECOSUR, Chetumal, Mexico.

Principal Component Analysis (PCA) was used to describe the relationship between the chemical variables measured in the water with each sampling location. The compiled data set representing the environmental variables analyzed in this study was transformed into a "site x variable" matrix. Euclidean distance and ordinations were plotted with FactoMineR and factoextra in Rstudio **[44]**.

### Biogeochemical analysis

The biogeochemical analyzes of the microbialite fabric were carried out with different methodologies. For total carbon and nitrogen we used a soil analyzer (Thermo Scientific Flash 2000). Barnard's calcimeter method **[45]** was used for inorganic carbon analysis by the determination of calcium carbonates. Total phosphorus was measured through solubilization by acid digestion (HNO_3_/HClO_4_). Available phosphorus was determined with the Olsen method **[46].** Organic matter and organic carbon were determined with Walkley and Black method **[47].** Determinations were done in the Soils and Plants Analysis Laboratory, ECOSUR, San Cristóbal.

### X-Ray Diffraction (XRD)

For XRD analyses samples were cold dried (10°C), homogenized with a pestle and agate mortar and sieved through a mesh < 75μm. The measurement was made in the angular interval 2θ from 5° to 80° in step scanner with a "step scan" of 0.003° and an integration time of 40 sec per step, using double-side aluminum holders (unoriented fractions). Each diffractogram was obtained in a diffractometer (Empyrean) equipped with a Ni filter, a monochromator, a thin tube focus copper and PIXcel3D detector. The diffraction patterns were analyzed with the HighScore software (version 4.5) with reference patterns from the ICDDPDF-2 and ICSD databases. All determinations were done in the X-Ray Diffraction Laboratory, Institute of Geology, UNAM.

### Total DNA extraction and 16S rDNA amplification

DNA extractions of microbialite samples (0.25 g) were done in triplicate using the DNeasy PowerSoil® Kit (Qiagen) following the manufacturer's instructions. Amplifications of the 16S rDNA V4 region were done following an established protocol **[48].** Each sample was amplified in three independent PCR reactions. PCR conditions were: 98°C for 30 s followed by 35 cycles of 95°C for 30 s, 52°C for 40 s, and 72°C for 90 s, and a final elongation step of 12 min at 72°C, then kept at 4°C. PCR products were pooled and purified with Ampliclean carboxyl-coated magnetic beads (NimaGen, NDL). The purified amplicon library was quantified with a QUBIT fluorometer (Promega, USA). The amplicon library with 20 ng/μl sample was sequenced on an Illumina MiSeq 2 x 300 platform (Yale Center for Genome Analysis, CT, USA).

### Analysis of Illumina 16S rDNA V4 sequences

The 16S rDNA V4 sequences of 90 samples of microbialites collected throughout Bacalar Lagoon, were deposited in the GenBank under BioProject PRJNA 550210. In addition, the data used during the analyses are available in the Open Science Framework: https://osf.io/zme9y/. Sequences were denoised, chimera and singletons were removed, then sequences were assigned into ASVs (Amplicon Sequence Variants) in QIIME2 (v.2018.6) **[49]** and truncated at position 200 with DADA2 **[50]** using the plugin *qiime dada2 denoised-paired*. ASVs representing less than 0.01% of the sequences across the dataset were eliminated. Taxonomy was resolved using the SILVA database (release 132–99% OTUs, 515–806 region), with the *feature-classifier classify-consensus-vsearch* (v2.9.0) plugin **[51]**. Mitochondrial and chloroplast sequences were filtered out from the feature table before rarefaction. Rarefaction was done at 10,000 ASVs per sample, resulting in the removal of 14 samples that had less than 9,000 sequences. The total dataset includes 90 samples for 15 sites.

After QIIME analyses, all sequence data were analyzed using multivariate correlational and ordination methods in the R statistical environment (version 3.6.2), for this, we used Phyloseq R [52]. We considered using the R markdown document that contains the complete commands for the analysis which is available here: https://github.com/YanezAlfredo/The-microbiome-microbialites-in-Bacalar-Lagoon-Mexico.git. The weighted Unifrac matrix was used to calculate the dissimilarity between the groups (*D*). The associations between environment and prokaryote community structure from different sites are shown using a constrained multidimensional scaling by Canonical Analysis of Principal Coordinates (CAP) based on weighted unifrac distance dissimilarity **[53]**. The differences between regions in the lagoon was analyzed using the PERMANOVA approach **[54]**, implemented in “vegan” as the ADONIS function using R package.

The ASV table was used to construct the biological matrix of genetic diversity based on 16S rDNA taxonomy. The alpha diversity indices such as species and Shannon index were calculated with the R package “vegan” [https://cran.r-project.org, https://github.com/vegandevs/vegan]. Wilcoxon tests were used to test for group differences in microbial diversity. A Venn diagram was created to compare the North-Center and South-Center regions obtained by unifrac weighted analysis, using the DrawVenn tool available online (http://bioinformatics.psb.ugent.be/webtools/Venn/).The total sum of-squares of the community composition matrix was partitioned into additive components of species (ASVs) to obtain their contributions to beta diversity (SCBD) and the local contributions of individual sampling units to beta diversity (LCBD) **[55]**. Following Legendre and De Cáceres **[56]**, we first transformed (Hellinger) the species abundance per site matrix and then we calculated multiple-site β-diversity indices (betapart) **[57]**; LCBD and SCBD indices were ran in adespatial **[58]**, ade4 **[59]** and with beta.div functions in “vegan” **[56].**

## Results

### The physicochemical environment surrounding microbialites in Bacalar Lagoon

**Fig 2:**
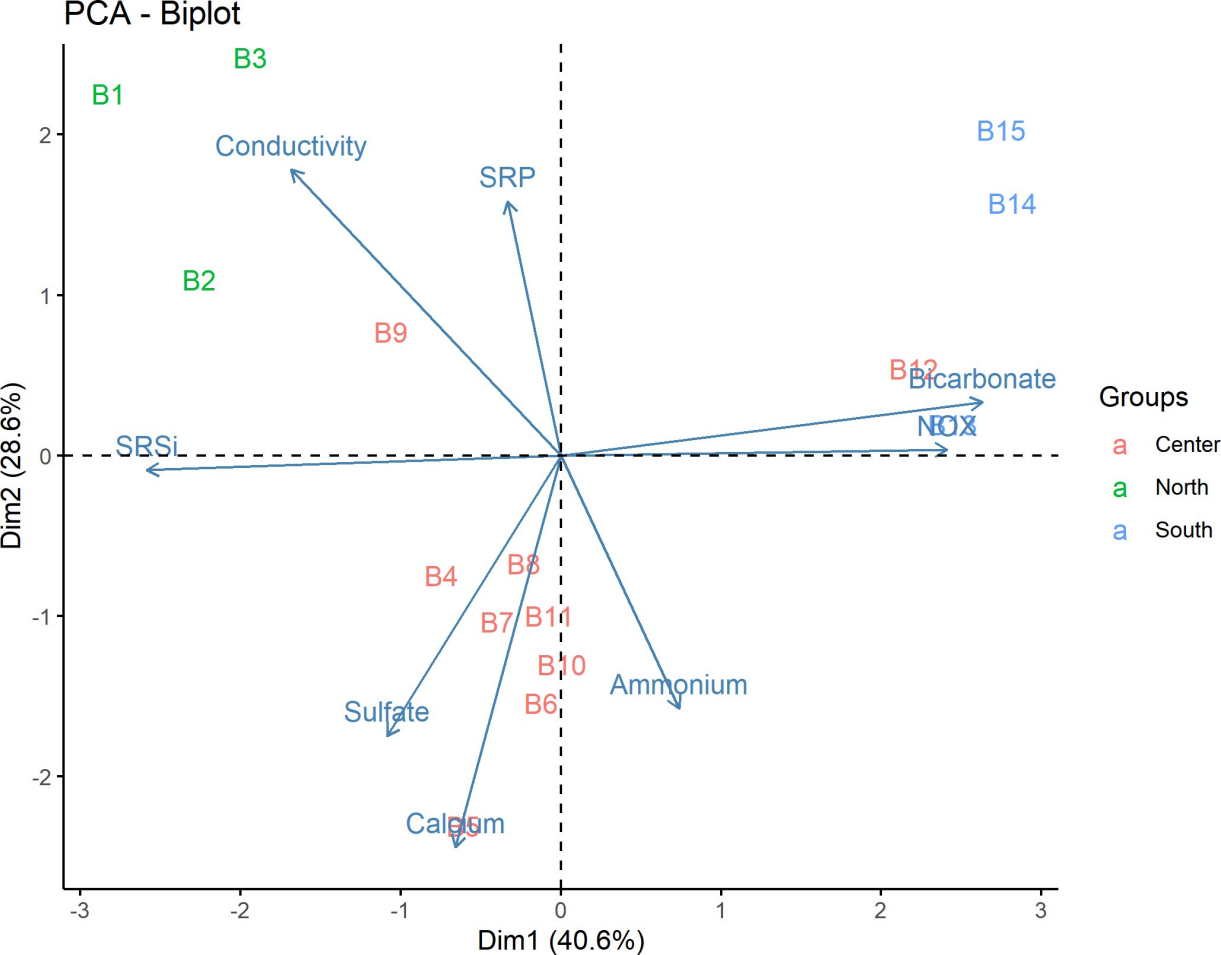
Spatial ordination (PCA) of environmental variables associated to microbialite sampling sites in Bacalar Lagoon.

The survey conducted in Bacalar Lagoon suggested an overall north-south gradient defined by higher conductivity and SRSi in the north, while the southern region had higher values of bicarbonate and available forms of nitrogen (ammonium, NOx), with similar values of sulfate and calcium throughout the lagoon (S1 Table). In the PCA, two general gradients were observed in Bacalar Lagoon. A north-south gradient based on PC1, where the following correlations were made: SRSi to HCO_3_^—^NOx (NO_3_^-^ + NO^-^_2_) with correlation coefficient values of -0.52 to 0.49, 0.52 respectively. The second gradient is interpreted on PC2, from the central zone towards the north with variables such as Ca-NH_4_^+^-SO_4_^2-^ to Conductivity-SRP, correlation coefficient values of -0.55, -0.042, -0.34 to 0.42, 0.45, respectively. None of the variables represented a strong component to explain the ordination (Fig 2).

Nonetheless, certain variables separated these regions, including bicarbonates, NOx (NO_3_^-^ + NO^-^_2_) and ammonium in the center-southern sampling sites (B 12–15) which increase near urban areas (Fig 2). Likewise, an analysis of previous research in the lagoon showed that concentrations of nitrates and ammonium increased two orders of magnitude between 2008 and 2018 in the southern sampling sites **[8, 30, 60, this study]** (Table 1).

**Table 1:** Available nutrient concentrations (nitrate, ammonia and soluble reactive phosphorus) in Bacalar Lagoon.

Region	Year	NO_3_^-^	NH_4_^+^	SRP	Reference
**South**	2008	0	0.036	BLD	Beltrán et al., 2012
**South**	2009	0.15	0.11	BLD	Centeno et al., 2012
**South**	2016	1.94	0.15	BLD	Tobón-Velázquez et al., 2018
**South**	2018	1.42	0.12	0.07	This study
**North**	2018	0.38	0.06	0.08	This study

The concentration of nutrients are presented in mg/l. BLD, below the limit of detection.

### Microbialite mineral and biogeochemical composition

Bacalar Lagoon microbialites were composed mainly of calcite (CaCO_3_) (~97%) and other minerals (3%) such as quartz (SiO_2_), siderite (FeCO_3_), kieserite (MgSO_4_) and ternadite (Na_2_SO_4_) (S2 Table). Regarding the biogeochemical characteristics of microbialites, we observed that no regional differences existed. All structures had similar values with respect to organic matter (om), nitrogen and carbon (S3 Table). The C:N ratio suggested a productive community.

### Microbialite genetic composition (16S rDNA V4)

A total of 4,167,392 reads were obtained for the 16S rDNA V4 hypervariable region. The mean number of sequences per site was 40,071. To include samples from all sites we defined a rarefaction at 10,000 sequences per subsample per site. All the microbialites were fully characterized at this sampling coverage.

**Fig 3:**
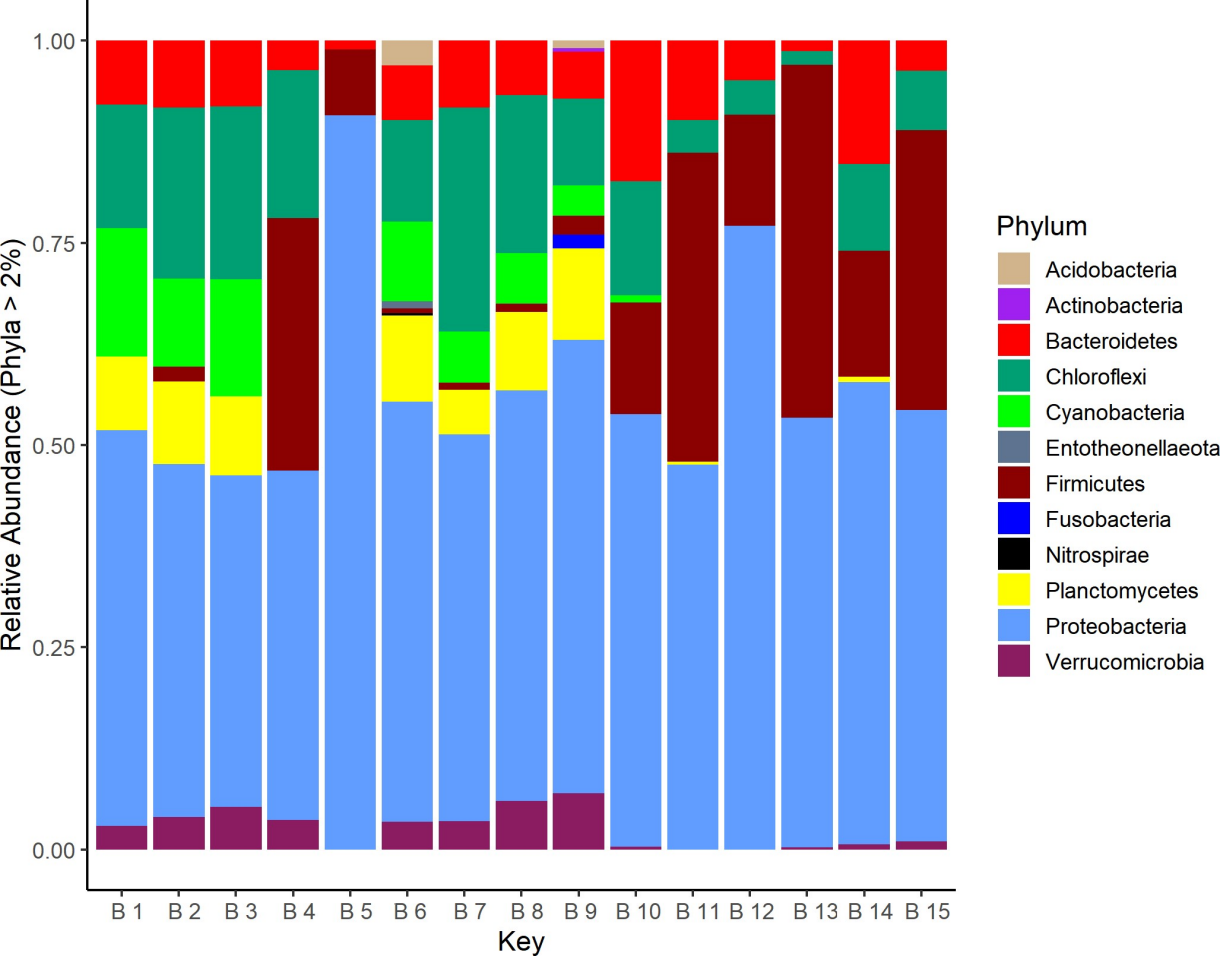
Microbialite bacterial genetic composition (16S rDNA V4) at the phylum level.

The prokaryote genetic composition at the phylum level indicates that 99.5% of all reads were assigned to Bacteria (Fig 3) and 0.5% to Archaea (S1 Fig). The main bacterial phyla showed great heterogeneity among sites: Proteobacteria (40–80%) was the most abundant, where class Gammaproteobacteria had the largest abundance at certain sites, (5–79%), followed by Alphaproteobacteria (14–25%) and Deltaproteobacteria (1–10%); Chloroflexi (7.6–14%); Cyanobacteria (1–11%); Firmicutes (1–23%); Bacteroidetes (7–8%); Planctomycetes (1–8%) and Verrucomicrobia (1–4%). Phyla with low abundances in all sites included Acidobacteria, Actinobacteria, Nitrospira, Chlamydiae, Spirochaetes, and Gemmatimonadetes.

**Fig 4:**
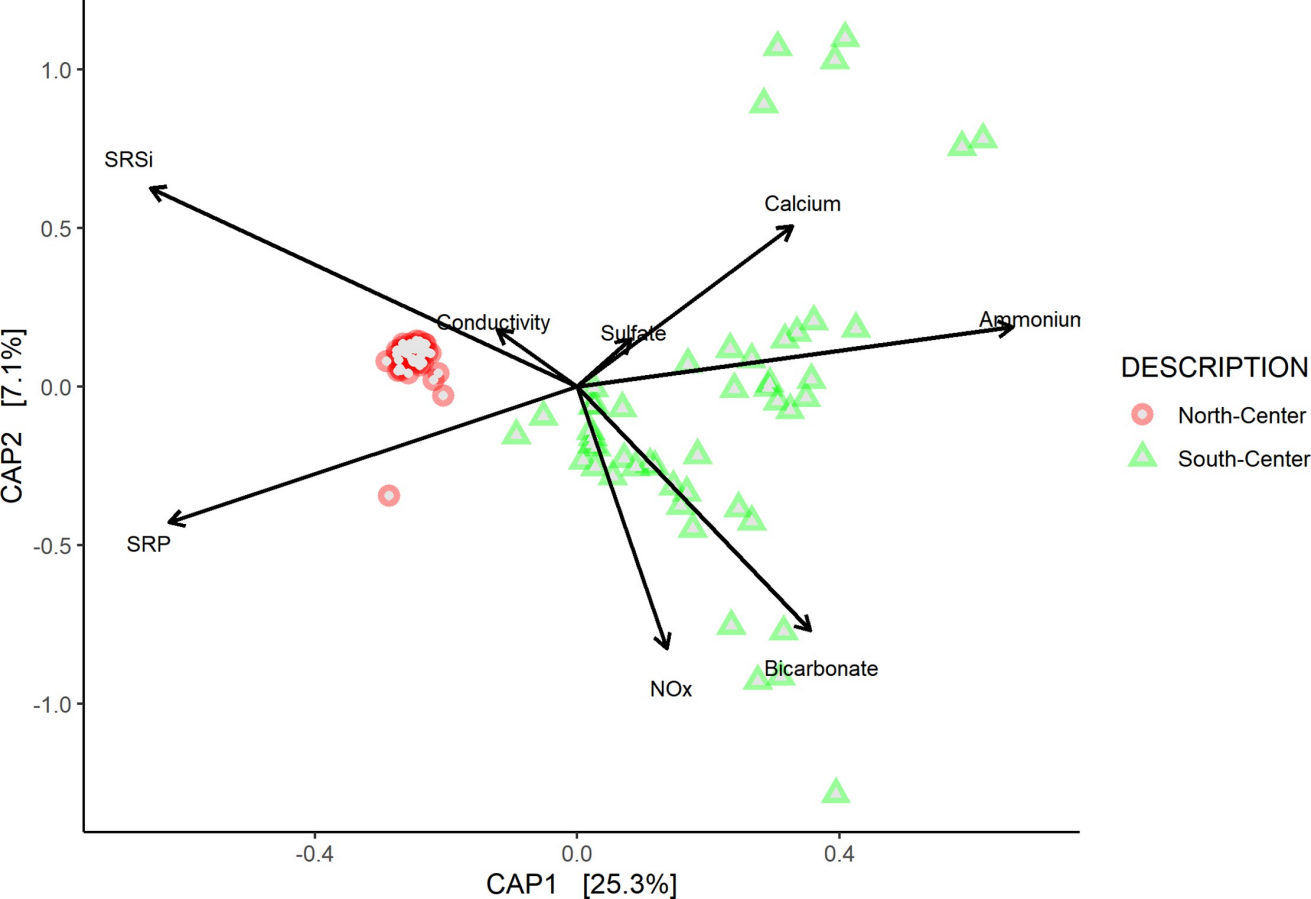
Constrained Analysis of Principal coordinates (CAP) based on Weighted-Unifrac and environmental variables.

The UniFrac weighted distance matrix separated Bacalar microbialites in two phylogenetically differentiated microbial communities. This result allowed us to classify the 15 sampling sites into two regions (S2 Fig). The first region was defined as North-Center and included sites B 1–3 and B 6–9, which represented 80% of the global microbial diversity and were very similar between them (D = 0.82). The second region was defined as South-Center with sites B 4–5 and B 10–15. Overall, the CAP of the genetic diversity matrix and environmental dataset, suggested that the factors that correlate in the South-Center region of Bacalar Lagoon with microbialite diversity are the concentrations of available forms of N (NO_3_^-^ and NH_4_^+^, respectively) (Fig 4). PERMANOVA analysis also indicated that the differences between regions in Bacalar Lagoon were significant (p<0.05).

**Fig 5:**
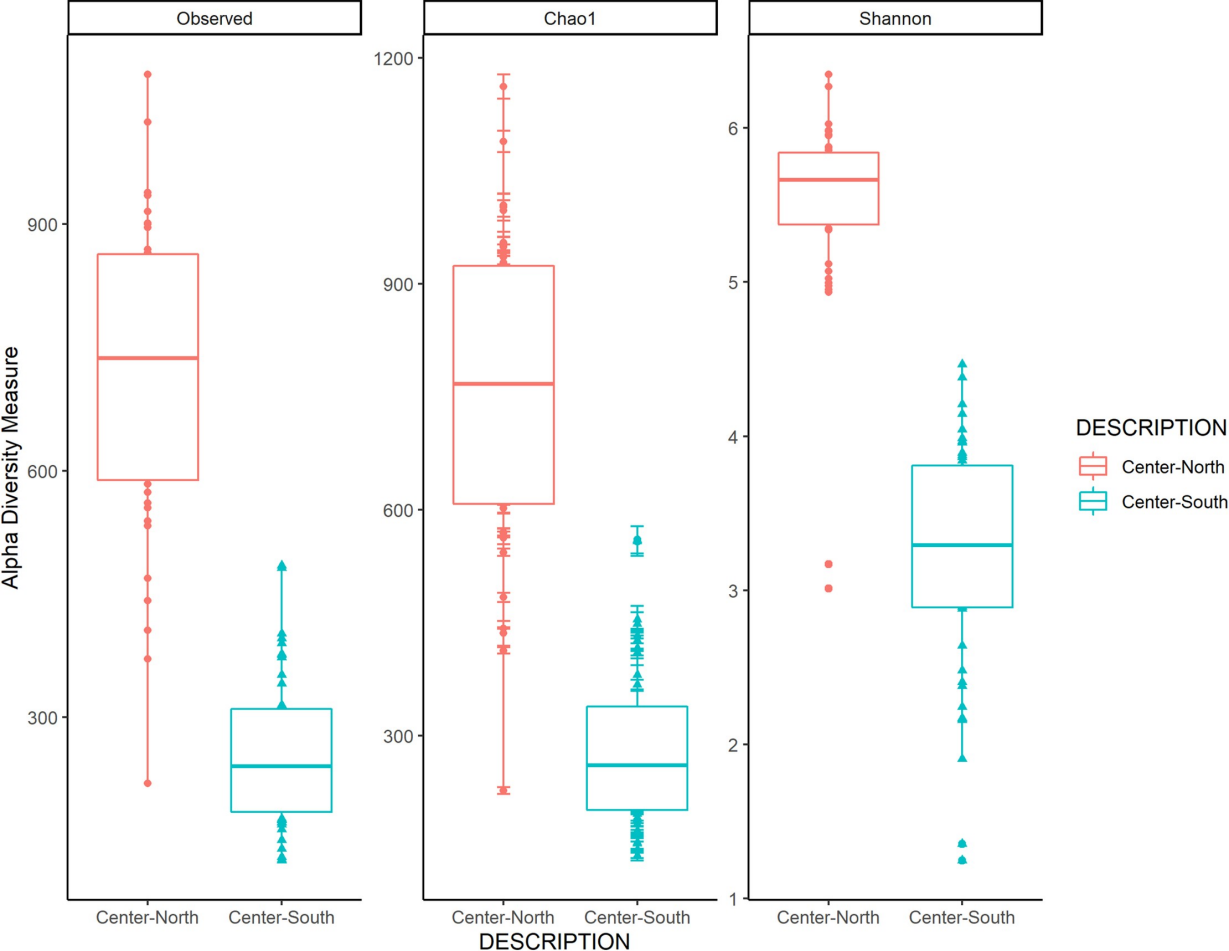
Observed counts and alpha diversity measured by the Chao1 and Shannon indices in the microbialites of Bacalar Lagoon: North-Center (sites B 1–3 and B 6–9) and South-Center (sites B 4–5 and B 10–15).

A Mann-Whitney-Wilcoxon test was conducted to compare the richness and diversity indices between the North-Center and South-Center regions. Several diversity indices demonstrated that the microbiome diversity of the North-Center was significantly greater than that observed in the South-Center region (p < 0.01) (Fig 5). The Shannon index indicated that the sampling sites North-Center of the town of Bacalar had a greater bacterial diversity (*H´* = 5.7), and the sites to the South-Center had 42% less diversity (*H'* = 3.3).

**Fig 6:**
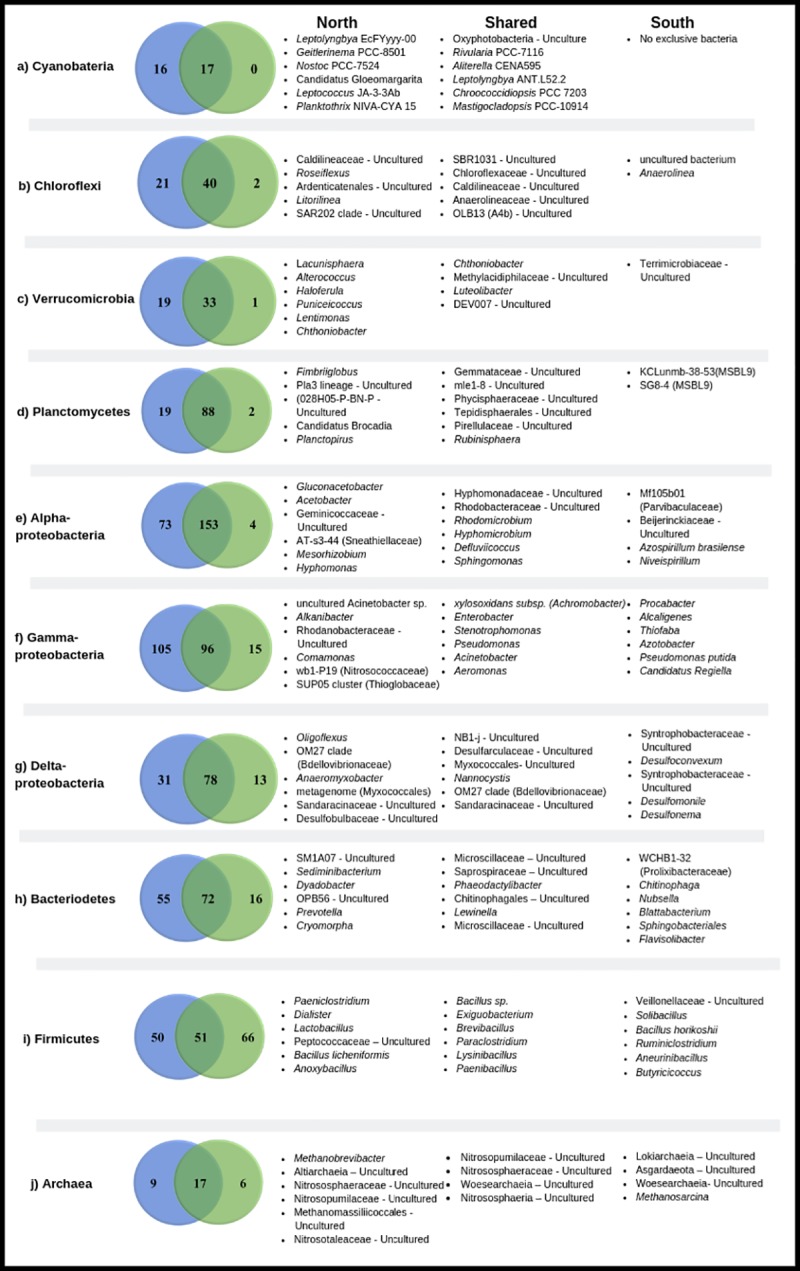
Venn diagram of main prokaryotes. (a) Cyanobacteria, (b) Chloroflexi, (c) Verrucomicrobia, (d) Planctomycetes, (e) Alphaproteobacteria, (f) Gammaproteobacteria, (g) Deltaproteobacteria, (h) Bacteroidetes, (i) Firmicutes, (j) Archaea.

The following groups defined microbialite bacterial diversity within the North-Center region in 66% of the relative abundance: Alphaproteobacteria (25%), Chloroflexi (14%), Deltaproteobacteria (10%), Cyanobacteria (11%), Bacteroidetes (8%) Planctomycetes (8%) and Verrucomicrobia (4%). On the other hand, the microbialites that develop in the South-Centerregion of Bacalar Lagoon, showed less abundance of bacterial groups, while 50% of the bacterial diversity was shared with their North-Center counterparts. Changes in composition between microbialites of both regions was characterized by a decrease in Alphaproteobacteria (14%), Chloroflexi (7.6%), Cyanobacteria (1%) and Deltaproteobacteria (1%) in the south. Bacteria that make up to 64% of the total diversity in microbialites were represented by Gammaproteobacteria (41%) and Firmicutes (23%) (Fig 3, Fig 6A–6J). Cyanobacteria, which are fundamental components of microbialites, shared 50% of their diversity between regions, with an average abundance of 10% for the North-Center and 1% for the South-Center. Shared cyanobacteria among all sites included Nostocales (*Calothrix*, *Rivularia*, *Scytonema*, *Nostoc*, *Mastigocladopsis*), Chroococcales (*Chroococcidiopsis*), Oscillatoriales (*Aliterella*, *Lyngbya*, *Leptolyngbya*, *Phormidium*). Cyanobacteria in the northern region had 16 exclusive species including *Calothrix*, *Geitlerinema*, *Gloeomargarita*, *Leptolyngbya*, *Nostoc*, *Oscillatoria* and *Scytonema*, among others, while the south did not show exclusive species (Fig 6A). Archaea have been reported as regular components of microbialites, yet their contribution is not fully understood. Archaea represented 0.5% and 0.2% of the total diversity in north and south microbialites, respectively. Six phyla (Altiarchaeota, Asgardaeota, Diapherotrites, Euryarchaeota, Nanoarchaeaeota and Thaumarchaeota) were identified in this study. Again, the microbialites in the North-Center had the greatest diversity, where Heimdallarchaeia, Woesearchaeia and Nitrososphaeria were the most abundant (Fig 6J).

To elucidate why these significant changes in community structure were occurring and which taxa were associated with variations at each site, we used the LCBD and SCBD metrics, as proposed by Legendre and De Cáceres [56]. The highest and most significant differences in LCBDs were found at sites B 5, B 12 and B 13 (p<0.05). The SCBD showed that *Pseudomonas*, *Aeromonas*, *Stenotrophomonas*, *Acinetobacter*, *Bacillus*, *Chryseobacterium*, *Achromobacter*, *Brevundimonas* and *Bacillus* were bacterial genera that contributed mostly to community structure substitution.

## Discussion

The Yucatan peninsula is an emerged carbonated continental platform. The northern region emerged during the Paleogene and Neogene, while the south began to rise in the Oligocene **[61].** There are no rivers in the Yucatan peninsula and karst features including channels and sinkholes (cenotes) are common **[62]**. In particular, the southeastern Yucatan peninsula is located in an evaporite region **[63]**, that has a high ecological connectivity and is the area where the greatest number of sites with microbialites are reported: Chetumal Bay **[64]**, Muyil (Sian Ka´an) and Bacalar Lagoon **[8, 30].**

The hydrogeochemical dynamics of Bacalar Lagoon are considered unique, with a high rate of constant exchange between the surface and groundwater flows **[40]**. The concentration of bicarbonate-NOx and the values of conductivity-SRSi were variables that defined a gradient in Bacalar Lagoon. There are sites around the world that host microbialites with hydrogeochemical characteristics similar to those of Bacalar Lagoon (carbonate saturation), such as Pavillon Lake in Canada; Great Salt Lake in the United States; Satonda in Indonesia **[27]**. The north zone of tBacalar is characterized by higher electrolytic conductivity, due to the connectivity with other lagoons such as Chile Verde, Salada and the Bay of Chetumal **[40, 65–66]**. The south of Bacalar, has higher bicarbonate concentration than the North and has higher concentrations of NOx **[30, 60]**. Sulfates are homogeneous throughout the lagoon, as described by Johnson et al., **[39]** and Beltrán et al., [30]. While, Sánchez et al., [66] reported that southern Quintana Roo has high rates of infiltration of nutrients—such as nitrates- and there is a high risk of contamination of the aquifer by human activities, such as agriculture.

Economic development and population growth are direct threats to freshwater ecosystems **[67–68]**. Nitrogen is often the limiting nutrient in aquatic marine environments, and P, in karst regions, is extremely low due to interactions with carbonate **[69]**. The concentration of ammonium ions and NOx is increasing in Bacalar Lagoon, especially near the city and south of the lagoon **[8, 39, 60,** this study]. The increasing presence of available forms nitrogen is one of the main causes of water quality change in freshwater bodies **[70].** We are observing a change in the natural oligotrophic conditions of Bacalar Lagoon. Other sites with increased eutrophication have shown that the productivity of the system alters the interactions of microbialites with eukaryotes, favoring competition the organisms such as algae, bivalves and diatoms **[71]**.

### Biogeochemical and mineralogical and characterization of Bacalar Lagoon microbialites

Microbialite are spatially distributed throughout Bacalar Lagoon. Bacalar Lagoon's hydrogeochemical dynamics make it different from other sites with microbialites in seawater and continental environments. All the microbialites analyzed in Bacalar Lagoon share mineral composition (CaCO_3_, ~97%). Valdespino et al., [23] reported a similar mineralogy for the microbialites of Bacalar Lagoon and Cuatro Cienegas Basin, which are water bodies of karstic origin. Bacalar Lagoon, which is located in the evaporative hydrogeochemical region **[62]**, presents carbonate dissolution processes of the subterranean water tunnels that reach the lagoon and the walls (carbonate rock) of the cenotes within the lagoon, favoring the saturation of bicarbonates **[37, 72]**. The development of larger microbialites in the south of Bacalar might be associated to bicarbonate saturation. Chagas et al., **[27]** also report for lacustrine systems with microbialites such as Lake Pavilion, Lake Van, Cuatro Cienegas Basin, Alchichica and Clifton, that calcite minerals and aragonite are the main minerals in microbialites.

Depending on the chemistry of the water and the bacterial community, microbialites present a diverse range of minerals, although generally they have been reported in greater percentage aragonite, hydromagnesite, gypsum and calcite **[23, 73].** Cyanobacteria such as Pleurocapsales and Chroococcales and Alphaproteobacteria are associated with the formation of aragonite in microbialites from Lake Alchichica (Mexico), a Mg-rich hyperalkaline crater lake (pH 8.9), while in Cuatro Cienegas and Bacalar, a S-rich karstic system, filamentous cyanobacteria and Sulfate Reducing Bacteria (SRB) favor calcite precipitation **[39]**. The hydrogeochemical conditions in Bacalar Lagoon favor the presence of bacterial groups (including cyanobacteria and S-cycling bacteria) that are involved in carbonate precipitation processes **[20, 22, 74]** and SRB reduce sulfates to sulfides with a consequent oxidation of organic carbon to bicarbonates. They contribute to a state of saturation, which occurs within the EPS matrix (associated mainly to cyanobacterial activity), precipitated by cyanobacteria in an alkaline pH, where calcium ions finally precipitate as CaCO_3_ **[33, 75]**. We report the same groups of SRB (Desulfovibrionales, Desulfobacteraceae, Syntrophobacteraceae, Desulfobulbaceae and *Desulfomonile*), distributed in all Bacalar Lagoon sites and reported by Johnson et al., [39]. All of these SRB have larger abundances in microbialites of the North-Center region.

In addition, Bacalar Lagoon microbialites have been described for their interactions with organisms such as gastropods (*Pomacea flagellata*), bivalves (*Mytilopsis sallei*), nematodes and mangroves **[38, 76].** Johnson et al., [39] reported the presence of Cyanobacteria and Rhizobiales, a nitrogen-fixing Alphaproteobacteria, in the microbialites associated with mangroves in their study sites in southern Bacalar Lagoon.

### Bacterial community structure of the microbialites of Bacalar Lagoon

This study proposes the presence of two phylogenetically differentiated communities in Bacalar Lagoon microbialites. Generally, studies mention that population differences occur in biogeographic patterns with the differentiation of niches at large geographic scales **[77]**. However, within ecosystems, biogeographic regionalization is possible due to the presence of gradients that induce changes in biological communities **[3].** Currently, anthropological activities can be considered a selective force, either physically (implementation of infrastructure) or by chemical alteration, which includes eutrophication of water bodies related to nutrient availability **[12]**.

The microbialite sites that represent the South-Center region of this study are located near the city of Bacalar, and to the south of the lagoon. These sites are associated to urban development in the shoreline of the lagoon which lack infrastructure for domestic water treatment, have leaking septic systems, agriculture and intense tourist activity, that are causing trophic affectation in the system **[68, 78–80].** Alterations in water quality was related to changes in the structure of the microbiome of microbialites between the North-Center and the South-Center regions Bacalar Lagoon. Recently, the work of Lindsay et al., **[13]** reported that in Great Salt Lake (GSL), USA, the bacterial community of microbialites responded to anthropogenic perturbation of the system related to construction of a railroad causeway. These authors demonstrated that microbialites in less disturbed areas of GSL have a greater abundance of cyanobacteria and diatoms compared to the almost total absence of these organisms in the microbialites where disturbance exists. Therefore, the monitoring of the community diversity of the microbialites, could be a strategy to know how bacterial groups react to the processes of alteration of the environment **[81]**, either before a physical affectation or through the chemical changes of the water as in the case of Bacalar Lagoon.

Microbialites in the world maintain, regardless of their geographical region, a similar composition at the phylum level **[8, 23].** Actinobacteria, Bacteroidetes, Cyanobacteria and Proteobacteria **[8, 37–39],** are common components of microbialites. Bacalar Lagoon microbialites in the North-Center region have a high diversity (*H´* = 5.7) (Fig 5), which contributes to understand that oligotrophy is not a limiting factor in the development of complex communities **[81].** The decrease of almost half of the bacterial diversity in the South-Center region is associated to dominance of specific microbes of the Gammaproteobacteria and Firmicutes groups.

Cyanobacteria were most abundant in the North-Center region. Considering that the South-Center region of Bacalar Lagoon is suffering an increase of nutrients due to anthropogenic activities, our results coincide with other works where it is reported that cyanobacteria are more diverse in oligotrophic waters than in eutrophic waters **[82]**. Cyanobacteria, a phylum that is relevant in EPS formation and has been considered to form nucleation sites for carbonate precipitation **[83–84],** showed a greater abundance and diversity in the North-Center microbialites of Bacalar Lagoon. The North-Center region also presented a higher diversity of Planctomycetes and Verrucomicrobia (~8.4% and ~3.8%, respectively), both forming part of a taxonomic super phylum called PVC **[85–86]**, described with a relative abundance between 7–12% in different microbialites of the world **[8, 86]**. Recently, the presence of these bacteria was correlated in places where calcite crystals predominated **[23]**. Chloroflexi, an anoxygenic phototrophic phylum, which participates in the "alkaline machinery" which in combination with oxygenic photosynthesis by cyanobacteria and sulfate reduction, promote the precipitation of carbonated minerals **[20, 36]**, was also more abundant in the North-Center region. This would suggest that loss of cyanobacterial, PVC and chloroflexi diversity could affect microbialite growth and maintenance in the South-Center region of Bacalar.

Further, the microbiome of microbialites in the South-Center region presented a high abundance of Firmicutes (~23.3%). This group occupies between 0–2% of relative abundance in other microbialites of the world **[87]**, and is thus, not common in healthy microbialite fabrics. Firmicutes generally have low percentages in oligotrophic water conditions and their abundance may suggest an environmental pollution processes **[82],** as reported for Gonghu Bay, China, where one of the causes of increased nutrients was domestic wastewater **[88]**. The class (eg. Bacilli) of the Firmicutes are used as indicators of fecal pollution in freshwater and their main sources are untreated domestic waters **[89]**, as may be happening in the South-Center region of our study.

### Changes in the bacterial community of microbialites in Bacalar Lagoon

It is important to define the factors that are causing the environmental disturbance of a system, especially if it is due to human activities **[90]**. We used the LCBD-SCBD metrics and a CAP to associate the environmental variants of home site (niche) and the association with their bacterial community (dispersion) **[91].** Legendre and De Cáceres [56] mention that high values of LCBD indicate the degree of ecological singularity of each sampling site. From this perspective, sites with high values of LCBD may contain unusual species or are sites that respond to human disturbance **[92]**. In both cases, the use of beta-diversity metrics can be a starting point for decision-making in conservation or ecological restoration scenarios **[93]**. In this study, the highest values of LCBD were related to sites in the South-Center region. The sites B5, B 12 and 13, obtained the highest LCBD values. Site B5 is a particular case of microbialite growth that has a strong correlation in the CAP to ammonium. Further research is needed to identify the sources of ammonium to this specific area in Bacalar Lagoon that shows an increase in domestic and tourist developments.

The Mexican Caribbean is an area whose economy depends mainly on tourism related to its natural resources **[60, 94]**. Particularly within Quintana Roo, places like Cancun and Playa del Carmen that have intense tourist activity, show affectations to the water quality of the underground aquifer systems and cenotes **[95–96]**. Currently, Bacalar presents an increase in tourist occupation. According to the Mexican Government office of statistics (INEGI-SecTur, 2019) Bacalar Lagoon received approximately 90,000 tourists in 2018, which was twice the amount of tourist visitation in 2017 (45,000). This phenomenon is likely to continue and the infrastructure to accommodate these visitors is not available. Tobón-Velázquez et al., [60] mentioned that the lack of infrastructure regulation from the government could result in the degradation of the water quality of Bacalar Lagoon, hence affecting the microbialites.

A direct correlation is reported between the most visited sites for tourists and the lowest prokaryote diversity. In addition, the sites with the lowest diversity that are located at the South-Center region of the lagoon, are the same sites that have been historically used for tourism. These results postulate that the changes in the microbiome of microbialites along Bacalar Lagoon are probably associated to a greater extent, with poor water quality due to high concentrations of ammonium and NOx **[80, 97].**

### Disturbance in oligotrophic water conditions affect the structure of microbiome in the microbialites of Bacalar Lagoon

Environmental problems in aquatic ecosystems related to nutrient enrichment are observed in different parts of the world **[79, 98].** In particular, in karstic environments (such as the Yucatan peninsula), where groundwater is flowing through fractures, and complex cave systems interconnect water bodies, such as lagoons and coastal environments **[99]**. Groundwater discharge has been identified as an important source of nutrients in many aquatic ecosystems of the peninsula **[100]**. It should be noted that all human activity in the peninsula (settlement, intensive fertilizer farming practices, deforestation, tourism, lack of wastewater treatment) has a direct impact on nearby water bodies **[101–102]**, and affects the structure of mangrove communities, coral reefs, sea grasses **[103]**, and microbialite diversity.

Understanding the changes in the structure of microbial communities is crucial, as this information may provide insights of the system and later be used as bioindicators for assessing environmental problems **[104]**. Currently, values for available SRP in Bacalar Lagoon remain close the detection limit **[30, 60],** but the different forms of available forms of nitrogen (NH_4_^+^, NO_3_^-^) are alarmingly increasing **[8, 60]** (Table 1). After an environmental disturbance, the possibility of a community of returning to its previous state will depend on its genetic and physiological diversity **[105],** yet so far, no research has demonstrated that microbialite communities can recover in the short term.

A hypothesis that rises from this study is that microbialites in Bacalar Lagoon have the same phylogenetic origin, yet disturbances in water quality detected in the South-Center region are causing loss of biodiversity. Another possible explanation is that high concentrations of carbonate present in the South-Center region, promote larger and faster microbialite growth, which is associated to a different community structure, differing from their North-Center counterparts. So far, we do not have elements to prove any of these open questions, but we do know that microbialites have fundamental biological constituents, where Cyanobacteria and bacteria associated to S-cycling are the main contributors to microbialite formation and growth. We still need to understand the dynamics of the communities that form microbialites, while trying to document their transformations in fragile habitats, like the tropical lagoon that is represented in this study. The increase in available forms of nitrogen is preoccupying to say the least since our research shows this is associated to lack of water treatment and planned agriculture in the region. How much can the native communities, represented in this study by microbialites, deal with the rate of change that human activities cause in the environment?

## Supporting information

S1 Table Physicochemical variables describing the water column where microbialites develop in Bacalar Lagoon, Mexico. (DOCX)

S2 Table Mineral composition of Bacalar Lagoon microbialites. (DOCX)

S3 Table Biogeochemical characterization of microbialites in Bacalar Lagoon. (DOCX)

S1 Fig PCoA showing weighted and unweighted Unifrac distributions of microbial diversity in Bacalar Lagoon microbialites. North-Center (red), South-Center (blue).
(TIF)

S2 Fig Class level diversity of Archaea from Bacalar microbialites defined with the V4 hypervariable region of the 16S rDNA. (TIFF)
