# Supplementary material for: The microbiome of modern microbialites in Bacalar Lagoon, Mexico
Source: PLoS One. 2020 Mar 25;15(3):e0230071. doi: 10.1371/journal.pone.0230071 (PMC7094828; doi:10.1371/journal.pone.0230071)
Supplement: S1 Table — (DOCX) [file pone.0230071.s001.docx]

Table S2. Physicochemical variables describing the water column where microbialites develop in Bacalar lagoon, Mexico.

| Site | NO_X_  (NO_3-_ + NO-_2_) | NH_4_^+^ | SRSi | SRP | Conductivity | Ca^2+^ * | HCO_3_^-^* | SO_4_^2-^ * |
| --- | --- | --- | --- | --- | --- | --- | --- | --- |
|  |  |  |  |  |  |  |  |  |
| B 1 | 2.1±0.08 | 4.3 ±0.02 | 463 ±0.00 | 0.10±0.00 | 4622 | 338 | 110 | 1170 |
| B 2 | 2.0±0.03 | 4.3 ±0.01 | 471 ±0.00 | 0.09±0.00 | 4436 | 338 | 110 | 1170 |
| B 3 | 1.7±0.01 | 0.9 ±0.05 | 470 ±0.00 | 0.10±0.00 | 4335 | 310 | 104 | 1030 |
| B 4 | 3.5±0.03 | 7.5 ±0.07 | 451 ±0.05 | 0.05±0.03 | 2950 | 388 | 120 | 1140 |
| B 5 | 1.4±0.00 | 6.9 ±0.01 | 446 ±0.09 | 0.08±0.02 | 2275 | 400 | 120 | 1140 |
| B 6 | 7.3±0.20 | 5.1 ±0.13 | 457 ±0.05 | 0.08±0.01 | 2043 | 404 | 140 | 1190 |
| B 7 | 8.4±0.01 | 4.3 ±0.05 | 463 ±0.01 | 0.11±0.00 | 2154 | 404 | 140 | 1190 |
| B 8 | 15.3±0.3 | 2.9 ±0.10 | 462 ±0.02 | 0.07±0.01 | 2280 | 404 | 140 | 1190 |
| B 9 | 4.0±0.20 | 3.7 ±0.17 | 464 ±0.01 | 0.09±0.01 | 2284 | 313 | 104 | 1154 |
| B 10 | 7.0±0.0 | 8.0 ±0.01 | 446±0.12 | 0.07±0.01 | 2468 | 404 | 140 | 1190 |
| B 11 | 8.0±0.10 | 6.5 ±0.50 | 448.±0.02 | 0.09±0.01 | 2482 | 404 | 140 | 1190 |
| B 12 | 13.9±0.3 | 8.0 ±0.30 | 422 ±0.07 | 0.06±0.01 | 2550 | 325 | 183 | 1072 |
| B 13 | 24.5±0.2 | 3.7 ±0.20 | 420 ±0.00 | 0.07±0.00 | 2132 | 329 | 220 | 1185 |
| B 14 | 22.7±0.3 | 4.8 ±0.05 | 426 ±0.05 | 0.08±0.01 | 2459 | 314 | 238 | 1038 |
| B 15 | 21.9±0.2 | 2.4 ±0.05 | 421 ±0.09 | 0.06±0.01 | 2580 | 309 | 232 | 1031 |
| The concentration of nutrients is expressed in µM/l. | | | | | | | | |
| Data with * was obtained from Gischler et al. 2008, values are in mg/l. | | | | | | | | |
| Conductivity is expressed in μS/cm | | | | | | | | |
