# Supplementary material for: The microbiome of modern microbialites in Bacalar Lagoon, Mexico
Source: PLoS One. 2020 Mar 25;15(3):e0230071. doi: 10.1371/journal.pone.0230071 (PMC7094828; doi:10.1371/journal.pone.0230071)
Supplement: S2 Table — (DOCX) [file pone.0230071.s002.docx]

Table S3. Mineral composition of Bacalar lagoon microbialites.

| Site | Calcite | Quartz | Siderite | Other  (Kisserite, Thenardite ) |
| --- | --- | --- | --- | --- |
| B 1 | 97 | 0 | 0 | 3 |
| B 2 | 97 | 0 | 0 | 3 |
| B 3 | 97 | 0 | 0 | 3 |
| B 4 | 97 | 0 | 0 | 3 |
| B 5 | 100 | 0 | 0 | 0 |
| B 6 | 97 | 0 | 0 | 3 |
| B 7 | 97 | 0 | 0 | 3 |
| B 8 | 97 | 0 | 0 | 3 |
| B 9 | 97 | 0 | 0 | 3 |
| B 10 | 100 | 0 | 0 | 0 |
| B 11 | 90 | 0 | 2 | 2 |
| B 12 | 100 | 0 | 0 | 0 |
| B 13 | 100 | 0 | 0 | 0 |
| B 14 | 98 | 1 | 1 | 0 |
| B 15 | 99 | 1 | 0 | 0 |
