# Supplementary material for: The microbiome of modern microbialites in Bacalar Lagoon, Mexico
Source: PLoS One. 2020 Mar 25;15(3):e0230071. doi: 10.1371/journal.pone.0230071 (PMC7094828; doi:10.1371/journal.pone.0230071)
Supplement: S3 Table — (DOCX) [file pone.0230071.s003.docx]

| Table S4. Biogeochemical characterization of microbialites in Bacalar lagoon. | | | | | | | |
| --- | --- | --- | --- | --- | --- | --- | --- |
| **Site** | **Pa** | **om** | **Nt** | **Ct** | **Cinorg** | **Corg** | **C:N** |
| B 1 | 0.0038 | 7.03 | 0.49 | 14.1 | 9.98 | 4.08 | 8.33 |
| B 2 | 0.0056 | 7.59 | 0.49 | 14.03 | 9.54 | 4.40 | 9.2 |
| B 3 | 0.0111 | 5.67 | 0.44 | 13.48 | 10.10 | 3.29 | 7.48 |
| B 4 | 0.0239 | 7.6 | 0.49 | 16.74 | 12.23 | 4.11 | 8.39 |
| B 5 | 0.0542 | 3.7 | 0.29 | 15.1 | 12.52 | 2.17 | 7.48 |
| B 6 | 0.0147 | 2.34 | 0.32 | 12.3 | 10.92 | 1.96 | 6.125 |
| B 7 | 0.0038 | 6.95 | 0.49 | 14.1 | 9.98 | 4.03 | 8.22 |
| B 8 | 0.0147 | 3.79 | 0.36 | 12.9 | 10.44 | 2.44 | 6.66 |
| B 9 | 0.0138 | 7.24 | 0.43 | 14.7 | 10.42 | 4.2 | 9.77 |
| B 10 | 0.0111 | 4.4 | 0.2 | 15.1 | 12.22 | 2.56 | 12.8 |
| B 11 | 0.0290 | 4.55 | 0.27 | 13.5 | 10.66 | 2.64 | 9.78 |
| B 12 | 0.0138 | 11.24 | 0.45 | 19.6 | 12.89 | 6.52 | 14.49 |
| B 13 | 0.0340 | 6.82 | 0.42 | 16.1 | 11.94 | 3.96 | 9.43 |
| B 14 | 0.0138 | 4.1 | 0.23 | 14.7 | 12.32 | 2.1 | 9.13 |
| B 15 | 0.0189 | 2.81 | 0.18 | 15.54 | 13.61 | 1.61 | 8.94 |
| The values are presented in mg/kg.  Pa = available phosphorus; om = organic matter; Nt = total nitrogen; Ct = total carbon; Cinorg = inorganic carbon; Corg = organic carbon; C:N = Corg:Nt ratio | | | | | | | |
