# Supplementary figures and images for: The microbiome of modern microbialites in Bacalar Lagoon, Mexico
Source: PLoS One. 2020 Mar 25;15(3):e0230071. doi: 10.1371/journal.pone.0230071 (PMC7094828; doi:10.1371/journal.pone.0230071)

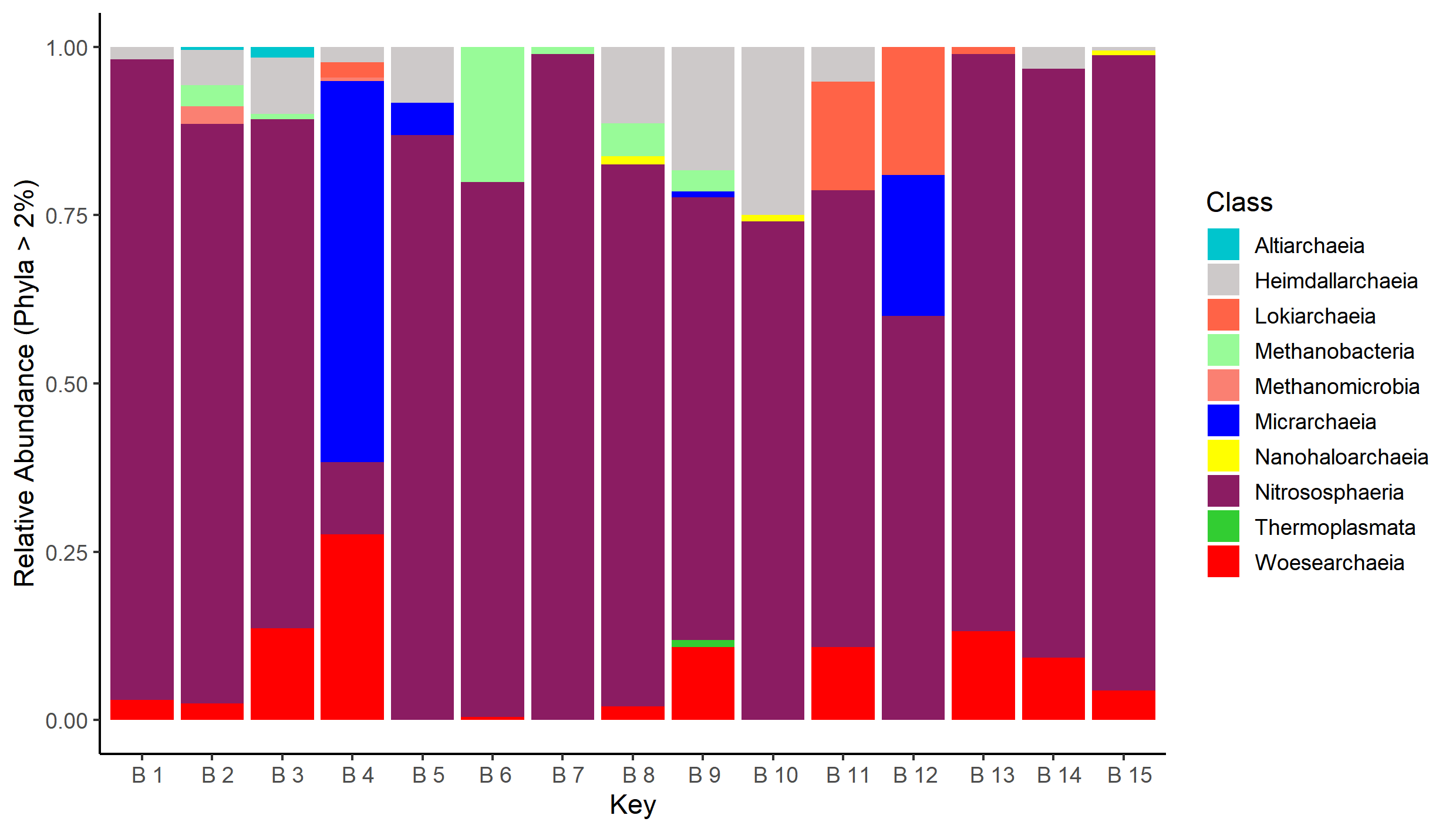

Supplement: S1 Fig — North-Center (red), South-Center (blue). (TIF) [file pone.0230071.s004.tif]

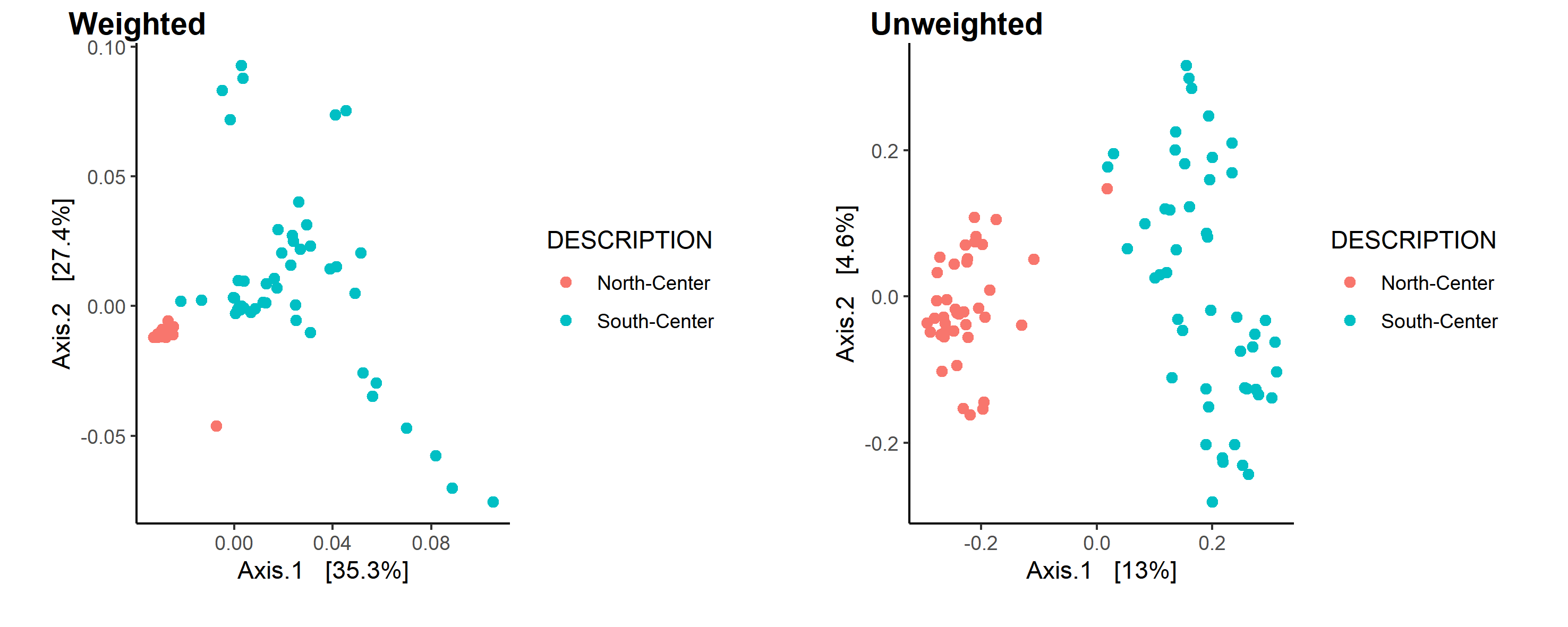

Supplement: S2 Fig — (TIFF) [file pone.0230071.s005.tiff]
